# Validated and longitudinally stable asthma phenotypes based on cluster analysis of the ADEPT study

**DOI:** 10.1186/s12931-016-0482-9

**Published:** 2016-12-15

**Authors:** Matthew J. Loza, Ratko Djukanovic, Kian Fan Chung, Daniel Horowitz, Keying Ma, Patrick Branigan, Elliot S. Barnathan, Vedrana S. Susulic, Philip E. Silkoff, Peter J. Sterk, Frédéric Baribaud, For the ADEPT (Airways Disease Endotyping for Personalized Therapeutics) and U-BIOPRED (Unbiased Biomarkers for the Prediction of Respiratory Disease Outcome Consortium) investigators, Ian Adcock, Nora Adriaens, Hassan Ahmed, Antonios Aliprantis, Kjell Alving, Charles Auffray, Philipp Badorrek, Per Bakke, David Balgoma, Aruna T. Bansal, Clair Barber, Frédéric Baribaud, An Bautmans, Annelie F. Behndig, Elisabeth Bel, Jorge Beleta, Ann Berglind, Alix Berton, Jeannette Bigler, Hans Bisgaard, Grazyna Bochenek, Michel J. Boedigheimer, Klaus Bøonnelykke, Joost Brandsma, Armin Braun, Paul Brinkman, Dominic Burg, Davide Campagna, Leon Carayannopoulos, Massimo Caruso, Rocha João Pedro Carvalho da Purificação, Amphun Chaiboonchoe, Romanas Chaleckis, Pascal Chanez, Kian F. Chung, Courtney Coleman, Chris Compton, Julie Corfield, Arnaldo D’Amico, Barbro Dahlen, Sven-Erik Dahlén, Jorge De Alba, Pim de Boer, Inge De Lepeleire, Betrand De Meulder, Tamara Dekker, Ingrid Delin, Patrick Dennison, Annemiek Dijkhuis, Ratko Djukanovic, Aleksandra Draper, Jessica Edwards, Rosalia Emma, Magnus Ericsson, Veit Erpenbeck, Damijan Erzen, Klaus Fichtner, Neil Fitch, Louis J. Fleming, Breda Flood, Stephen J. Fowler, Urs Frey, Martina Gahlemann, Gabriella Galffy, Hector Gallart, Trevor Garrett, Thomas Geiser, Julaiha Gent, de Verdier Maria Gerhardsson, David Gibeon, Cristina Gomez, Kerry Gove, Neil Gozzard, Yi-ke Guo, Simone Hashimoto, John Haughney, Gunilla Hedlin, Pieter-Paul Hekking, Elisabeth Henriksson, Lorraine Hewitt, Tim Higgenbottam, Uruj Hoda, Jens Hohlfeld, Cecile Holweg, Ildiko Horvath, Peter Howarth, Richard Hu, Sile Hu, Xugang Hu, Val Hudson, Anna J. James, Juliette Kamphuis, Erika J. Kennington, Dyson Kerry, Matthias Klüglich, Hugo Knobel, Richard Knowles, Alan Knox, Johan Kolmert, Jon Konradsen, Maxim Kots, Linn Krueger, Norbert Krug, Scott Kuo, Maciej Kupczyk, Bart Lambrecht, Ann-Sofie Lantz, Lars Larsson, Nikos Lazarinis, Diane Lefaudeux, Saeeda Lone-Latif, Matthew J. Loza, Rene Lutter, Lisa Marouzet, Jane Martin, Sarah Masefield, Caroline Mathon, John G. Matthews, Alexander Mazein, Sally Meah, Andrea Meiser, Andrew Menzies-Gow, Leanne Metcalf, Roelinde Middelveld, Maria Mikus, Montse Miralpeix, Philip Monk, Paolo Montuschi, Nadia Mores, Clare S. Murray, Jacek Musial, David Myles, Shama Naz, Katja Nething, Ben Nicholas, Ulf Nihlen, Peter Nilsson, Björn Nordlund, Jörgen Östling, Antonio Pacino, Laurie Pahus, Susanna Palkonen, Ioannis Pandis, Stelios Pavlidis, Giorgio Pennazza, Anne Petrén, Sandy Pink, Anthony Postle, Pippa Powel, Malayka Rahman-Amin, Navin Rao, Lara Ravanetti, Emma Ray, Stacey Reinke, Leanne Reynolds, Kathrin Riemann, John Riley, Martine Robberechts, Amanda Roberts, Graham Roberts, Christos Rossios, Anthony Rowe, Kirsty Russell, Michael Rutgers, Thomas Sandström, Giuseppe Santini, Marco Santoninco, Corinna Schoelch, James P. R. Schofield, Wolfgang Seibold, Dominick E. Shaw, Ralf Sigmund, Florian Singer, Marcus Sjödin, Paul J. Skipp, Barbara Smids, Caroline Smith, Jessica Smith, Katherine M. Smith, Päivi Söderman, Adesimbo Sogbesan, Ana R. Sousa, Doroteya Staykova, Peter J. Sterk, Karin Strandberg, Kai Sun, David Supple, Marton Szentkereszty, Lilla Tamasi, Kamran Tariq, John-Olof Thörngren, Bob Thornton, Jonathan Thorsen, Salvatore Valente, Aalderen Wim van, Marianne van de Pol, Drunen Kees van, Geest Marleen van, Jenny Versnel, Jorgen Vestbo, Anton Vink, Nadja Vissing, Garnier Christophe von, Ariane Wagener, Scott Wagers, Frans Wald, Samantha Walker, Jonathan Ward, Zsoka Weiszhart, Kristiane Wetzel, Craig E. Wheelock, Coen Wiegman, Siân Williams, Susan J. Wilson, Ashley Woodcock, Xian Yang, Elizabeth Yeyasingham, Wen Yu, Wilhelm Zetterquist, Koos Zwinderman

**Affiliations:** 1 Immunology Therapeutic Area, Janssen Research & Development LLC, 1400 McKean Rd, Spring House, 19477 USA; 2 NIHR Respiratory Biomedical Research Unit, Clinical and Experimental Sciences, Faculty of Medicine, University of Southampton, Southampton, UK; 3 National Heart and Lung Institute, Imperial College & Biomedical Research Unit, Royal Brompton & Harefield NHS Trust, London, UK; 4 Academic Medical Centre, University of Amsterdam, Amsterdam, The Netherlands

**Keywords:** Cluster analysis, Biological markers, Observational study

## Abstract

**Background:**

Asthma is a disease of varying severity and differing disease mechanisms. To date, studies aimed at stratifying asthma into clinically useful phenotypes have produced a number of phenotypes that have yet to be assessed for stability and to be validated in independent cohorts. The aim of this study was to define and validate, for the first time ever, clinically driven asthma phenotypes using two independent, severe asthma cohorts: ADEPT and U-BIOPRED.

**Methods:**

Fuzzy partition-around-medoid clustering was performed on pre-specified data from the ADEPT participants (*n* = 156) and independently on data from a subset of U-BIOPRED asthma participants (*n* = 82) for whom the same variables were available. Models for cluster classification probabilities were derived and applied to the 12-month longitudinal ADEPT data and to a larger subset of the U-BIOPRED asthma dataset (*n* = 397). High and low type-2 inflammation phenotypes were defined as high or low Th2 activity, indicated by endobronchial biopsies gene expression changes downstream of IL-4 or IL-13.

**Results:**

Four phenotypes were identified in the ADEPT (training) cohort, with distinct clinical and biomarker profiles. Phenotype 1 was “mild, good lung function, early onset”, with a low-inflammatory, predominantly Type-2, phenotype. Phenotype 2 had a “moderate, hyper-responsive, eosinophilic” phenotype*,* with moderate asthma control, mild airflow obstruction and predominant Type-2 inflammation. Phenotype 3 had a “mixed severity, predominantly fixed obstructive, non-eosinophilic and neutrophilic” phenotype, with moderate asthma control and low Type-2 inflammation. Phenotype 4 had a “severe uncontrolled, severe reversible obstruction, mixed granulocytic” phenotype, with moderate Type-2 inflammation. These phenotypes had good longitudinal stability in the ADEPT cohort. They were reproduced and demonstrated high classification probability in two subsets of the U-BIOPRED asthma cohort.

**Conclusions:**

Focusing on the biology of the four clinical independently-validated easy-to-assess ADEPT asthma phenotypes will help understanding the unmet need and will aid in developing tailored therapies.

**Trial registration:**

NCT01274507 (ADEPT), registered October 28, 2010 and NCT01982162 (U-BIOPRED), registered October 30, 2013.

**Electronic supplementary material:**

The online version of this article (doi:10.1186/s12931-016-0482-9) contains supplementary material, which is available to authorized users.

## Background

Asthma is a disease driven by complex and heterogeneous pathobiologic processes, involving a multitude of inflammatory and structural cell types and a large number of pro-inflammatory and tissue remodeling mediators [1]. This heterogeneity, may explain, at least in part, the severity of the disease, including varying risk of exacerbations [2] and the inconsistency in responses observed across the spectrum of asthma patients to both standard therapies and the emerging biologics [3, 4].

Despite a plethora of published studies on asthma mechanisms, the definition of asthma remains limited to the description of its key clinical features, with broad reference to the underlying inflammatory characteristics and heterogeneity. This is true for both general definitions provided by the Global Initiative for Asthma guidelines (GINA, http://www.ginasthma.org/) and definitions of its severe forms [1], while stratification of asthma is still based on the combination of symptoms, lung function and treatment required for symptom control. Recognizing that clinical and pathobiologic features do not follow a linear, incremental pattern presented in the GINA guidelines, a number of studies of asthmatics with varying clinical presentations have applied unbiased clustering in an attempt to define new phenotypes of asthma. These studies have used a range of clinical variables in combination with simple, easy to apply, measures of airways inflammation assessed in induced sputum [5–8]. Indeed, the Severe Asthma Research Program (SARP) clustered on clinical, demographic, and natural history variables from 726 asthmatics, resulting in 5 patient clusters [5]. Subsequently, in a subset of these subjects (*n* = 423), 15 inflammatory cellular measures and clinical variables were included, and 4 clusters were identified [6]. In a recent SARP report, the dataset was reduced (*n* = 378) to participants with exhaled nitric oxide (FENO) and bronchoalveolar lavage (BAL) fluid cell counts, and included healthy controls, resulting in 6 clusters by clustering on 112 clinical, physiologic, and inflammatory variables [7]. Patient data from the Dose Ranging Efficacy And safety with Mepolizumab (DREAM) study was clustered on clinical and biomarker variables [8]. Four clusters were identified that could be defined by 3 predictors (blood eosinophils, airway reversibility, and body mass index). However, neither the longitudinal stability of the described clusters nor their validation in an independent asthma cohort has reported to date. Furthermore, limited studies to date have undertaken an in-depth characterization of the molecular processes that are associated with the clinical phenotypes. Such analysis is limited to a single study of mild, steroid-naive asthmatics by Woodruff and colleagues [9] who assessed gene transcription in the airways of 42 nonsmoking subjects with asthma, 28 nonsmoking healthy controls, and 16 current smokers without asthma but with mild to moderate airflow obstruction (disease controls) and observed two broad clusters defined by the expression of three genes (POSTN, CLCA1 and SERPINB2) induced by the type-2 (T2) cytokine interleukin (IL)-13. The two clusters, defined by high and low expressions levels of these three genes, were termed Th2-high and Th2-low, the latter composed of asthmatic and healthy individuals.

The current study had two main aims. The first aim was to identify clinical phenotypes defined by a limited set of clinical variables that are easily acquired and can, therefore, be used in routine clinical practice or trials, and to assess their reproducibility and stability over time. The second aim was to describe these clinical phenotypes by their molecular characteristics, as assessed by whole genome expression of bronchial samples acquired by bronchoscopic biopsies and brushings. The study used clinical and biomarker data from two independent cohorts. Initial clustering that created the clinical phenotypes was performed on clinical data acquired in the ADEPT (Airway Disease Endotyping for Personalized Therapeutics) study [10] involving 158 asthmatics. The phenotypes thereby produced were assessed for stability within the longitudinal arm of the ADEPT study and validated for reproducibility using data from a subset of asthmatics from the U-BIOPRED (Unbiased Biomarkers for the Prediction of Respiratory Disease Outcome) study [11] constituting an independent validation cohort.

## Methods

### Study design and study populations

The ADEPT and U-BIOPRED study designs and study populations are published elsewhere [10, 11] and are described briefly here. The current study consisted of a combination of cross-sectional (ADEPT and U-BIOPRED) and longitudinal (ADEPT) studies. Participants in both cohorts were assessed clinically at baseline, using pre-specified protocols.

Clinical phenotypes were created by clustering 9 clinical variables (see Clustering methodology in the next paragraph) from the ADEPT study which served as the training set. Matching datasets from U-BIOPRED, excluding airway hyperresponsiveness (AHR), which was not assessed in U-BIOPRED, were used as the validation set for the ADEPT study-derived clinical phenotypes. After 3, 6 and 12 months of follow up, the ADEPT study participants were further reassessed to enable further validation, i.e. assessment of the stability of the phenotypes through classification of the followed up participants into the ADEPT study-derived phenotypes created with baseline data. Relevant biological samples were collected to define the pathobiologic (transcriptomic) characteristics of the clinical phenotypes. Both the ADEPT [10] and U-BIOPRED [11] studies collected serum for protein array analysis. All participants underwent bronchoscopy for differential gene expression analysis by microarray of endobronchial biopsies (in ADEPT) and epithelial brushings (U-BIOPRED) (reasons for using different sample types are described below under “Airway type-2 inflammation high and low phenotype”). All ADEPT participants underwent sputum induction at the screening visit, to fulfil inclusion criteria, and again at the Baseline Visit, to confirm the stability of their phenotype, while the U-BIOPRED participants had a single sputum induction.

Detailed inclusion and initial clinical stratification criteria of the two studies are published elsewhere [10, 11]. Briefly, for recruitment purposes, the 158 asthmatic participants in the ADEPT cohort were initially classified into severity groups based on their use of asthma controller medication and lung function as assessed by forced expiratory volume in one second (FEV_1_): mild (no asthma controller medications, FEV_1_ > 80% of predicted) (*n* = 52), moderate (low-moderate dose ICS, FEV_1_ 60-80% of predicted) (*n* = 55), or severe (on high-dose ICS, FEV_1_ 50-80% of predicted) (*n* = 51). FEV_1_ ranges were selected to reflect appropriate medication level (e.g., if a patient had FEV_1_ < 60% of predicted, a low-medium ICS dose may not be appropriate), with a low-bound in the severe group for safety considerations. All had been current non-smokers for at least one year, with <10 pack-year smoking history. The 530 asthmatics from the U-BIOPRED cohort were initially classified as non-severe (low-medium dose ICS) (*n* = 88), non-smoking severe (high-dose ICS; current non-smokers with <5 pack-year smoking history) (*n* = 110), and smoking severe (high-dose ICS; current or ex-smokers with at least 5 pack-year smoking history) (*n* = 311). All the asthmatics on maintenance oral corticosteroids (OCS) in the U-BIOPRED cohort were classified as severe. A subset of the U-BIOPRED cohort only was included in this study using those participants without missing data for the 8 clustering variables described below (*n* = 397 of 509) (see Additional file 1: Table S2). The 397 U-BIOPRED participants included both participants on maintenance OCS and smokers, two groups of participants which were not represented in ADEPT.

Clinical phenotypes were created by clustering 9 clinical variables (see Clustering methodology in the next paragraph) from the ADEPT study which served as the training set. Matching datasets from U-BIOPRED, excluding airway hyperresponsiveness (AHR), which was not assessed in U-BIOPRED, were used as the validation set for the ADEPT study-derived clinical phenotypes. After 3, 6 and 12 months of follow up, the ADEPT study participants were further reassessed to enable further validation, i.e. assessment of the stability of the phenotypes through classification of the followed up participants into the ADEPT study-derived phenotypes created with baseline data. Relevant biological samples were collected to define the pathobiologic (transcriptomic) characteristics of the clinical phenotypes. Both the ADEPT [10] and U-BIOPRED [11] studies collected serum for protein array analysis. All participants underwent bronchoscopy for differential gene expression analysis by microarray of endobronchial biopsies (in ADEPT) and epithelial brushings (U-BIOPRED) (reasons for using different sample types are described below under “Airway type-2 inflammation high and low phenotype”. All ADEPT participants underwent sputum induction at the screening visit, to fulfil inclusion criteria, and again at the Baseline Visit, to confirm the stability of their phenotype, while the U-BIOPRED participants had a single sputum induction.

### Clustering methodology

**Fig. 1:**
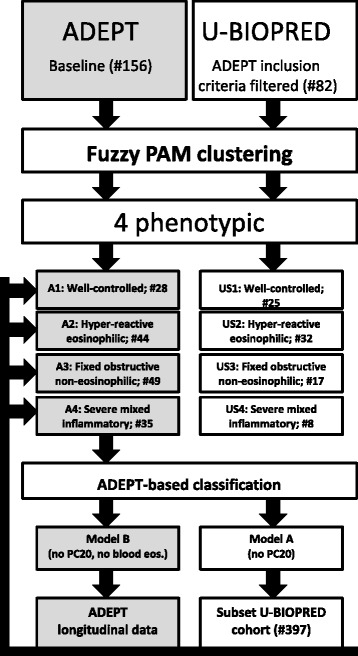
Schematic of clustering analyses. Fuzzy PAM clustering was used on 156 ADEPT and 82 U-BIOPRED asthma patients, defining analogous phenotypes, A1 to A4 for ADEPT and US1 to US4 for U-BIOPRED. GLMnet classification models for the ADEPT phenotypes was built and applied to either the ADEPT longitudinal samples (3, 6 and 12 month) or a large subset of the U-BIOPRED cohort (*n* = 397)

Figure 1 describes the overall schematic of clustering analyses. Initial clustering of the clinical data from the independent ADEPT (#156 of 158) and U-BIOPRED (#82 of 509) cohorts was performed using the Fuzzy Partition-around-Medoid (PAM) clustering method [12] to create the baseline phenotypes. For the purpose of validation of the identified phenotypes, the GLMnet-classification model of ADEPT-asthma baseline clinical clusters (#154 of 158) was then applied to classify the ADEPT-asthma subjects using the data from the baseline and 3, 6, and 12 month follow-up visits and the baseline data of U-BIOPRED participants (#397 of 509). The discrepancies between the cohort sizes and the number of patients used were due to missing data for various clustering variables.

Nine clustering variables were selected because they can be readily measured in standard clinics: pre-bronchodilator forced expiratory volume in 1 s (pre-BD FEV_1_) and forced vital capacity (FVC) expressed as % predicted, FEV1/FVC ratio, bronchodilator reversibility (BDR), airway hyper-responsiveness (AHR), i.e. log-transformed provocative concentration of methacholine resulting in 20% decline in FEV_1_ from baseline (PC_20_); Asthma Control Questionnaire (ACQ-7) [13], Asthma Quality of Life Questionnaire (AQLQ) [14]; log-transformed Fractional Exhaled Nitric Oxide (FENO) concentration in exhaled breath, and blood eosinophil counts expressed as absolute counts per μL.

PAM clustering is moderately robust to missing data, allowing the PC20 variable to be utilized despite a minority of ADEPT-asthma patients (20 of 158) not having values for this variable (methacholine challenge was not performed if FEV1 was <60% predicted). Two subjects in the mild asthma cohort were excluded because they did not have valid screening or baseline pre-bronchodilator spirometry measurements available, resulting in not having values for 4 of the 9 variables. Two ADEPT asthma subjects were missing baseline data for FENO and were included in the initial clustering but excluded from GLMnet classification analyses, described below. Therefore, 156 ADEPT asthma subjects were included in the initial clustering, and 154 for GLMnet classification analyses.

### Initial clustering

The Fuzzy Partition-around-Medoid (PAM) clustering method [12] (software: NCSS v8, www.NCSS.com; NCSS LLC, Kaysville, Utah) used Euclidean distance, scaled with average absolute deviation, and applied the ‘fuzzifier constant’ set to 1.1 (value selected to optimize Silhouette and distance metrics). The number of clusters selected for further analysis was based on maximizing between cluster distance (normalized Dunn’s partition coefficient, (Fc(U)) and minimizing within-cluster distance (normalized Kaufman’s distance partition coefficient, (Dc(U))) metrics [12]. The fuzzy clustering algorithm assigns to each subject a probability of membership to each of the clusters, with the subject then assigned to the cluster with the highest probability of membership.

Fuzzy PAM was selected because it is robust to outliers and missing data, does not make the assumption that all participants cleanly belong to a single cluster and because it has advantages over other standard methods, such as k-means clustering and hierarchical clusters, as well as traditional PAM. Because of limited sample sizes, permutation-based methods to identify stable clusters were not employed. Sensitivity analyses were performed to assess the robustness of these initial clusters in ADEPT (removing the medoid subjects and whole study arms), the longitudinal stability of the clusters, and the homologous clusters observed when clustering on U-BIOPRED subjects, overcome the limitations of not employing permutations.

For clustering of ADEPT participants, all 158 asthmatics from the mild, moderate, and severe study arms were included, with 2 mild asthmatics excluded because they did not have valid screening or baseline pre-bronchodilator spirometry measurements available. For clustering of U-BIOPRED participants, clustering was performed using the same methods and variables as ADEPT but excluding PC20 because this variable was available only in a minority of U-BIOPRED subjects. Participants with missing data for any of the 8 clustering variables or those taking maintenance OCS were also excluded to ensure the most robust clustering (*n* = 82 of 509 total participants, with additional exclusion of 194 on maintenance OCS, 64 smoking severe asthmatics, 148 with FEV_1_ out-of-range, and additional 21 with missing data).

### GLMnet classification

The GLMNet multinomial logistic regression classification algorithm (R package GLMNet v1.9-8 7 [15], http://www.jstatsoft.org/v33/i01/) was used with the R-package Caret (Classification and Regression Training, v. 6.0-35; http://caret.r-forge.r-project.org/) for model cross-validation (leave-one-out cross-validation to identify optimal GLMNet alpha and lambda tuning parameters).

Two models were used. Model A, the optimal classification model for ADEPT-asthma baseline clusters (Model A), based on the 8 clinical clustering variables (excluding PC20), was applied to classify U-BIOPRED subjects (*n* = 397, including patients on OCS and smokers), limited only to those without missing data for these 8 clustering variables. PC20 was not included in the classification because this variable was not longitudinally assessed in ADEPT and was unavailable for most U-BIOPRED participants. An alternate classification model, Model B, was built for longitudinal assessment of ADEPT asthma subjects (based on 3, 6, and 12 month data), excluding blood eosinophil counts (in addition to excluding PC20) because this variable was only measured at screening in ADEPT. To classify ADEPT subjects across the 3, 6 and 12 month visits and U-BIOPRED subjects into the determined clinical clusters based on the variables used in the original clustering of screening/baseline values for ADEPT-asthma subjects. Subjects were excluded from the classification analyses if they had missing data for at least one of the model variables at that time point.

The linear predictor coefficients for Model A and Model B needed to calculate probabilities of classification to the ADEPT baseline clinical cluster groups are reported in Additional file 1: Table S3. The probability for classification of subject *i* to outcome class *k* = *c* (of K outcome classes) is determined by first calculating the set of linear predictors ƒ(k,i) for subject *i* at each outcome class *k*: *f*(*k*, *i*) = ∑_*m* = 0_^*M*^(*βm*, *k* ⋅ ***X***_*m*,*i*_), where β_m,k_ is the linear predictor function coefficient for predictor *m* at outcome class *k* (with the coefficient at predictor m = 0 is the constant for the linear predictor function for class *k*) and ***X***_m,i_ is the value of predictor *m* for subject *i*.

The probability for classification of subject *i* to outcome class *c* [*Pr(Y*_*i*_ *= c)*] is the ratio of the exponentiated linear predictor for outcome class *c* over the sum of the exponentiated linear predictors across the K linear predictors for subject i: Pr(*Y*_*i*_ = *c*) = *e* ^*f*(*c*,*i*)^/∑_*k* = 1_^*K*^ *e* ^*f*(*k*,*i*)^. The outcome class with the highest probability for subject *i* is the outcome class assigned to the subject.

An interactive calculator (file: ADEPT_clinical_cluster.Classification_calculator.Model_A.xlsx) allowing readers to input values for the 8 clustering variables, with the ADEPT clinical cluster assignment and classification probabilities automatically calculated for ‘Model A’.

### Biologic sample acquisition and analysis

#### Bronchoscopic sampling and transcriptomic analysis

Endobronchial biopsies and epithelial brushings, taken at bifurcations of sub-segmental airways in the lower lobes, were immediately preserved in RNAlater® solution and then maintained at −70 °C [10, 11]. RNA was extracted using Qiagen miRNeasy kit (Qiagen; Germantown, MD) and amplified with NuGen ovation pico WTA kit (NuGen Technologies; San Carlos, CA). The cDNA was analyzed using the Affymetrix HG-U133 + PM microarray platform (Affymetrix, Santa Clara, CA). CEL files were normalized, assessed for quality control to exclude technical outliers (chip image analysis, Affymetrix GeneChip QC, RNA degradation analysis, distribution analysis, principal components analysis, and correlation analysis), and re-normalized using the robust multi-array (RMA) method. The log_2_-normalized data matrix was imported into OmicSoft ArrayStudio software (Cary, NC; www.omicsoft.com) for subsequent analysis. For ADEPT biopsies and U-BIOPRED brushings, batch effects from RNA processing sets were observed (2 sets for each sample type), with the batch effect adjusted in the data matrices using linear modeling of batch (as random factor) and cohort. A log_2_-intensity threshold of 5.5 for ADEPT biopsies and 4.75 for U-BIOPRED brushings was established as the limit of reliable quantification (LOD) based on the 90^th^ percentile signal of merged nonspecific probesets distribution in the array and by the inflection point of maximum variance with decreasing signal in a standard deviation vs. mean intensity plot across all probesets. Probesets with mean log_2_ intensity above this threshold in at least one of the 4 study cohorts were considered quantifiable and included in subsequent analyses (24033 and 21363 probesets for biopsies and brushings, respectively).

#### Induced sputum (IS) sampling and analysis

The sputum induction and processing protocols are published in detail elsewhere [10, 11, 16]. They differed in respect of duration of induction (maximum three 7-min sessions of nebulization in ADEPT and four 5-min sessions in U-BIOPRED) and concentration of hypertonic saline (increasing concentrations of 3, 4, and 5% for ADEPT and 0.9 to 4.5% for U-BIOPRED).

For both cohorts, mucoid portions of sputum were selected and treated with dithiothreitol for this study in all participants [17]. Sputum supernatant and cytospin slides for differential cell counts were prepared by standard methods. Standard differential staining and counting was performed centrally. For U-BIOPRED, assessments of a maximum of 400 inflammatory cells on Diff-Quick stained cytospins were performed centrally with the outcome of the cytospin analysis. Sample viability ≥50% and a threshold of ≤ 40% squamous cells was the default for samples being made available for analysis. For both studies, only samples with squamous cell content ≤30% from cytospin differential counts were included in the analyses. For ADEPT, a significant proportion of subjects had only a screening or only a baseline sample available that passed quality control standards. Therefore, the mean (differential cell counts) or geometric mean (analyte, gene expression measurements) of screening and baseline measurements was used for subsequent analyses.

#### Serum sampling and analysis

Serum was collected using standard Serum Separation Tubes (SST), frozen within 30 min and subsequently used for quantification of 1129 serum analytes applying the SomaScan v3 platform. Results for serum total immunoglobulin E (IgE) are presented from this panel, defining high IgE levels as those above the 95^th^ percentile of the HV distribution. In previous evaluations of the platform in asthmatics and HV, IgE measurements highly correlated (Pearson’s correlation coefficient r > 0.9) with those obtained from standard ELISA-based assays (data not shown).

### Airway type-2 inflammation high and low phenotype

For ADEPT, airway Th2-high status was defined on the basis of biopsy CCL26 gene expression (from microarray data) beyond the healthy control distribution, which also coincided with the limit of reliable quantification (LOQ) for the CCL26 probeset. Periostin (POSTN) gene expression (from microarray data) beyond the healthy control distribution was evaluated as an additional indicator of T2-high status. Endobronchial brushings samples from ADEPT were limited in sample size and therefore not applicable for assigning airway Th2-high status for subjects across the clinical clusters.

For U-BIOPRED, endobronchial brushings were selected as the airway sample type to evaluate Th2-high status because this was the sample type with the largest sample size and most overlap with other sample types (biopsies, sputum). Because CCL26 expression was below LOQ for most subjects across the study cohorts in U-BIOPRED biopsies and was also below LOQ in the brushings, an alternative Th2-activity indicator was selected, namely our own IL-13 *ex vivo* stimulation gene signature (IL-13 IVS) in endobronchial epithelial air-liquid interface cultures. The signature (genes in signature listed in Additional file 1: Table S4) was defined by selecting genes commonly induced by IL-13 stimulation across 3 independent sets of experiments (data not shown). Enrichment was evaluated on a per-subject basis using the R-Bioconductor package Gene Set Variation Analysis (GSVA, v 1.14.1) [18]. Subjects with enrichment scores (ES) beyond the 95^th^ percentile of healthy control distribution were classified as airway T2-high.

### Phadiatop® testing

The ImmunoCAP Phadiatop test (USA or Europe regional test, depending on the patients’ respective regions) (http://www.phadia.com/en-GB/5/Products/ImmunoCAP-Assays/1/) [19] was used to determine the atopic status of patients. ImmunoCAP Phadiatop is a blood test in which results are expressed as positive or negative. A positive Phadiatop**®** result indicates that the patient is atopic. A negative result indicates that the patient is non-atopic, i.e. not sensitized to inhalant allergens.

## Results

### The ADEPT clinical phenotypes

**Fig. 2:**
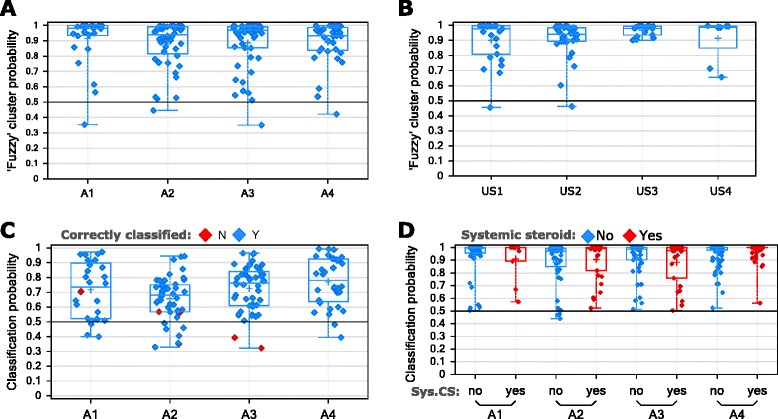
Probability of cluster membership. The probability of cluster membership for the assigned cluster (i.e., the cluster with maximum probability) output from the Fuzzy-PAM clustering algorithm is reported for each subject from **a** ADEPT-asthma cohorts (baseline) and **b** U-BIOPRED adult asthma cohorts. The classification probability from the GLMnet classification model A of the 8 clustering variables (excluding PC20 variable) is reported for (**c**) ADEPT asthma subjects (baseline), with discordantly classified subjects shown with red symbols, and **d** U-BIOPRED asthma, stratified for systemic corticosteroid use (blue, no; red, yes)

**Fig. 3:**
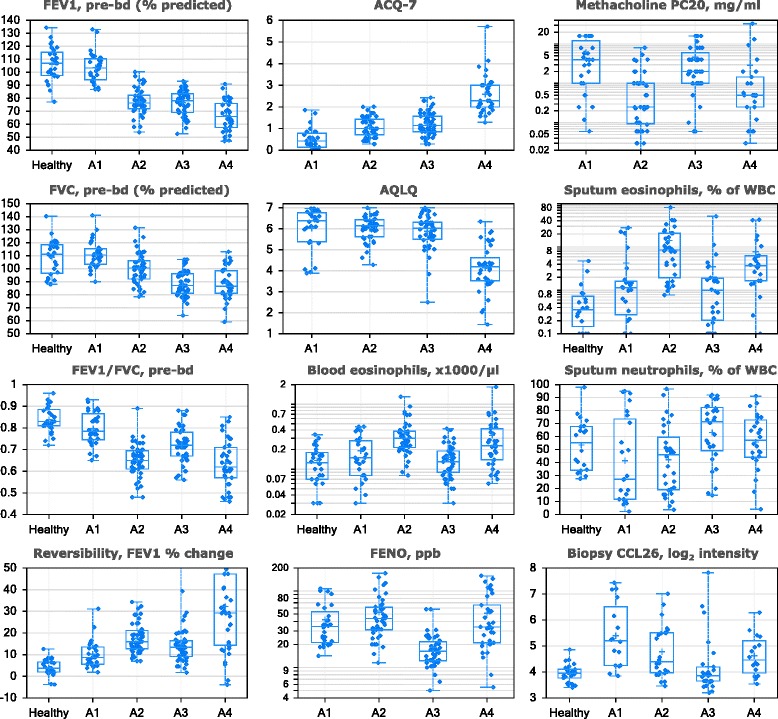
Clustering variables, sputum granulocytes, and biopsy CCL26 distributions in ADEPT clinical phenotypes. The values (y-axis) for the indicated variables (indicated at top of the plot) are shown for ADEPT asthma participants stratified by fuzzy-PAM clinical clusters (x-axis). Data presented as symbols representing individual participants and summarized by box (inter-quartile range and median) & whiskers (range), with ‘+’ indicating the mean. PreBD, pre-bronchodilator; WBC, white blood cells

**Fig. 4:**
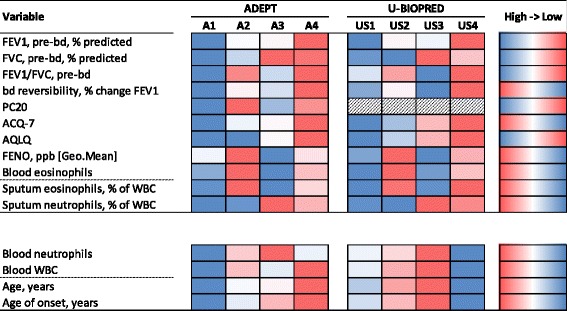
Mean values of clustering and sputum granulocyte variables among clinical clusters. Relative mean values of the indicated variables are schematically represented for each clinical cluster from ‘best’ (blue) to ‘worst’ (red) values among clusters within the indicated study (coloring for high-to-low values of variable indicated in right-most column)

The ADEPT asthmatics (*n* = 156) were partitioned into clusters, i.e. clinical phenotypes, based on baseline data of the 9 clustering variables. Partitioning the asthma population into four clusters was determined to be optimal based on maximizing between-cluster Silhouette metric Fc(U) and minimizing within-cluster distance metric Dc(U). The Fc(U)/Dc(U) values for options with 2, 3, 4, and 5 clusters were: 0.742/0.110, 0.768/0.080, 0.786/0.079 (optimal), and 0.772/0.082, respectively. >75% of participants in each cluster had >80% probability for their assigned phenotype, and only one subject per phenotype had <50% probability of ‘belonging’ to their phenotype (Fig. 2). The clinical and biomarker characteristics of the 4 ADEPT phenotypes (Clusters A1 to A4) are shown in Fig. 3 and Table 1 and graphically summarized in Fig. 4.

**Table 1:** Clinical, biomarker, and demographic characteristics of ADEPT clusters at baseline

Variable^a^	Healthy (*n* = 31)	Cluster A1 (*n* = 28)	Cluster A2 (*n* = 44)	Cluster A3 (*n* = 49)	Cluster A4 (*n* = 35)	*P*-value
Mean [Geo.Mean] ± SD						
FEV1, pre-bd, % predicted	106.1 ± 13.5	103.5 ± 11.8	77.4 ± 10.2	76.5 ± 9.8	66.8 ± 11.5	**<10** ^**−6**^
FVC, pre-bd, % predicted	108.8 ± 13.6	111.1 ± 10.8	100.0 ± 12.0	88.2 ± 9.8	88.7 ± 12.0	**<10** ^**−6**^
FEV1/FVC, pre-bd	0.84 ± 0.06	0.80 ± 0.08	0.65 ± 0.08	0.72 ± 0.08	0.64 ± 0.11	**<10** ^**−6**^
bd reversibility, % change FEV1	4.1 ± 3.6	10.2 ± 6.3	17.6 ± 7.0	14.8 ± 9.6	32.2 ± 21.9	**<10** ^**−6**^
ACQ7	na	0.5 ± 0.5	1.1 ± 0.5	1.2 ± 0.5	2.6 ± 0.9	**<10** ^**−6**^
AQLQ	na	6.0 ± 1.0	6.0 ± 0.6	5.9 ± 0.8	4.1 ± 1.1	**<10** ^**−6**^
FENO, ppb [Geo.Mean]	na	35.2 + 28.1/-15.6	43.8 + 36.7/-20.0	16.4 + 9.7/-6.1	37.7 + 47.8/-21.1	**<10** ^**−6**^
Blood eosinophils, ×1000/ul [Geo.Mean]	0.112 + 0.106/-0.054	0.150 + 0.168/-0.079	0.299 + 0.234/-0.131	0.131 + 0.100/-0.057	0.237 + 0.286/-0.130	**<10** ^**−6**^
PC20, mg/ml [Geo.Mean]	na	2.8 + 11.3/-2.2	0.3 + 1.4/-0.3	2.2 + 7.0/-1.7	0.6 + 2.8/-0.5	**<10** ^**−6**^
Blood neutrophils, ×1000/ul [Geo.Mean]	3.47 + 1.50/-1.05	3.26 + 1.03/-0.78	3.83 + 1.56/-1.11	3.93 + 1.65/-1.16	3.73 + 1.29/-0.96	0.0999
Blood WBC, ×1000/ul [Geo.Mean]	6.01 + 1.72/-1.34	6.01 + 1.43/-1.16	6.42 + 2.04/-1.55	6.32 + 2.12/-1.59	6.49 + 1.38/-1.14	0.6676
Sputum neutrophils, % of WBC^b^	53.6 ± 20.1	41.4 ± 33.0	42.2 ± 25.8	63.1 ± 23.8	55.9 ± 21.7	**0.0053**
Sputum eosinophils, % of WBC [Geo.Mean]^b^	0.4 + 0.8/-0.3	1.0 + 4.7/-0.8	7.1 + 18.7/-5.1	0.9 + 3.5/-0.7	3.5 + 10.5/-2.6	**<10** ^**−6**^
Serum IgE, RFU [Geo.Mean]	1.0 + 2.1/-0.7	7.6 + 29.5/-6.0	12.6 + 27.5/-8.6	7.1 + 20.3/-5.3	14.4 + 33.2/-10.0	0.0388
Age, years	31.5 ± 9.1	32.5 ± 14.3	42.4 ± 12.0	43.0 ± 11.8	46.8 ± 13.3	**0.0002**
Age of onset, years	na	15.4 ± 13.9	21.0 ± 14.0	22.7 ± 15.2	23.7 ± 18.6	0.1537
Body Mass Index (kg/m^2^)	24.7 ± 3.3	25.2 ± 3.2	25.7 ± 3.6	26.9 ± 3.7	26.6 ± 4.1	0.1622
Percent of clinical cluster						
Mild, no ICS (%)	na	93%	16%	27%	11%	
Moderate, low-medium ICS (%)	na	7%	52%	43%	26%	**<10** ^**−6**^
Severe, high ICS (%)	na	0%	32%	31%	63%	
Male (%)	65%	46%	48%	43%	43%	0.9500
Atopic, Phadiatop positive (%)	0%	79%	91%	69%	71%	**0.0670**
Serum IgE-high, >95th %ile of controls (%)	0%	71%	86%	69%	83%	**0.1692**
Sputum Pauci (Eos < %3, PMN < 60%) (%)^b^	45%	48%	16%	36%	21%	
Sputum Neutr. (Eos < 3, PMN > =60%) (%)^b^	50%	30%	13%	43%	17%	**0.0012**
Sputum Mix (Eos ≥ 3%, PMN > =60%) (%)^b^	0%	0%	13%	14%	29%	
Sputum Eos. (Eos ≥ 3%, PMN < 60%) (%)^b^	5%	22%	59%	7%	33%	
FENO ≥ 35 ppb (%)	na	50%	61%	4%	44%	**<10** ^**−6**^
Blood eosinophils ≥ 300/ul (%)	6%	21%	50%	6%	34%	**2.9 x10** ^**−5**^
FENO ≥ 35 ppb OR Blood eos. ≥ 300/ul (%)	na	57%	84%	10%	57%	**<10** ^**−6**^
Biopsy CCL26-high (%)^c^	0%	56%	42%	16%	31%	0.0515

^a^Summary statistics (mean, or geometric mean where indicated, and standard deviation; or percent of clinical cluster) and *p*-values from F-test (for mean/geomtric mean statistics) or x-square statistics for associations among the clinical clusters (not included healthy control cohort) are presented. The 9 clinical and clinical biomarker variables used in the clustering are presented first, followed by additional clinical, biomarker and demographic variable groupings

^b^For sputum differentials, *n* = 20, 23, 32, 38, and 24 for Healthy cohort and Clusters 1, 2, 3, and 4, respectively

^c^For endobronchial biopsy gene expression variables (from Affymetrix HG-U133 + PM array signal intensities), *n* = 25, 27, 28, 33, and 18 for Healthy cohort and Clusters 1, 2, 3, and 4, respectively

Significant *p* values are captured in bold

*Phenotype A1 (‘mild, normal lung function, early onset, low inflammation’)* comprised mostly mild asthmatics (mean ACQ-7: 0.5) and the vast majority (93%) were not currently on ICS. This was the phenotype with the lowest mean age-of-onset (15 yrs), BDR and AHR, and preserved lung function that was not different from healthy participants. Their inflammatory burden was low, based on mean FENO levels, the second lowest blood and sputum eosinophil counts, and the lowest sputum neutrophil counts.

*Phenotype A2 (‘moderate, mild reversible obstruction, hyper-responsive, highly atopic, eosinophilic’)* contained mostly moderate (52%) and to a lesser degree severe asthmatics (32%), as defined at the time of recruitment, characterized by mild, reversible airflow obstruction, and moderate asthma activity (mean ACQ-7: 1.1). However, this phenotype had the most hyper-responsive asthmatics, as measured by methacholine PC_20_, with the highest degree of eosinophilic inflammation (based on FENO and blood and sputum eosinophils), and they were the most atopic (based on total and specific serum IgE (Phadiatop®)).

*Phenotype A3 (‘mixed severity, mild reversible obstruction, non-eosinophilic, neutrophilic’)* consisted of a mix of asthmatics with mild (27%), moderate (43%), and severe (31%) disease. The majority were reasonably well-controlled and had mild airflow obstruction and AHR, less BDR (approximately 1/3 participants irreversible) and reduced FVC. Cluster A3 had the lowest FENO, the least eosinophilic inflammation, the lowest rate of atopy, but the highest sputum neutrophils.

*Phenotype A4 (‘severe uncontrolled, severe reversible obstruction, mixed granulocytic)* asthmatics were derived predominantly from the severe (63%) and to a lesser extent moderate (26%) asthmatics. They were generally the most severe and uncontrolled, with the greatest airflow obstruction, highest BDR, and high degree of AHR and were characterized by prominent mixed eosinophilic/neutrophilic inflammation. Although this phenotype had similar high proportions of serum IgE-high participants as Phenotype A2, the rate of atopy (Phadiatop® test, see Methods) was lower, similar to Cluster A3.

All 9 clustering variables significantly varied across the 4 ADEPT phenotypes as shown in Fig. 3 and Table 1, which report additional clinical, biomarker, and demographic variables. When restricting analysis to moderate and severe asthma, the 9 clustering variables also significantly varied across Phenotypes A2, A3, and A4 (Table 2).

**Table 2:** Clinical, biomarker, and demographic associations among the ADEPT moderate-severe asthma subjects in baseline clinical cluster groups

Variable^a^	Phenotype A2 (*n* = 37)	Phenotype A3 (*n* = 36)	Phenotype A4 (*n* = 31)	*P*-value
Mean [Geo.Mean] ± SD				
FEV1, pre-bd, % predicted	74.6 ± 8.2	73.8 ± 9.8	65.5 ± 11.5	**3.0 x10** ^**−4**^
FVC, pre-bd, % predicted	97.4 ± 9.8	87.4 ± 10.2	88.6 ± 12.2	**2.0 x10** ^**−4**^
FEV1/FVC, pre-bd	0.64 ± 0.08	0.70 ± 0.07	0.62 ± 0.10	**9.0 x10** ^**−4**^
bd reversibility, % change FEV1	17.3 ± 6.6	16.5 ± 10.5	32.0 ± 21.5	**1.1 x10** ^**−5**^
ACQ-7	1.1 ± 0.4	1.3 ± 0.6	2.6 ± 0.9	**<10** ^**−6**^
AQLQ	6.0 ± 0.6	5.8 ± 0.9	4.1 ± 1.2	**<10** ^**−6**^
FENO, ppb [Geo.Mean]	42.0 + 35.9/-20.9	15.6 + 8.8/-7.9	37.1 + 47.9/-17.8	**<10** ^**−6**^
Blood eosinophils, ×1000/ul [Geo.Mean]	0.317 + 0.244/-0.134	0.124 + 0.094/-0.037	0.235 + 0.285/-0.151	**<10** ^**−6**^
PC20, mg/ml [Geo.Mean]	0.5 + 1.8/-0.5	2.2 + 8.8/-2.2	0.4 + 1.4/-0.4	**1.0 x10** ^**−4**^
Blood neutrophils, ×1000/ul [Geo.Mean]	3.80 + 1.64/-3.80	3.95 + 1.78/-3.95	3.67 + 1.33/-3.67	0.6916
Blood WBC, ×1000/ul [Geo.Mean]	6.34 + 2.10/-0.35	6.35 + 2.28/-0.29	6.44 + 1.39/-0.44	0.9687
Sputum neutrophils, % of WBC^b^	43.1 ± 26.6	61.6 ± 25.1	56.2 ± 22.1	**0.0239**
Sputum eosinophils, % of WBC [Geo.Mean]^b^	7.8 + 20.7/-0.6	0.7 + 2.7/0.0	3.1 + 8.5/-0.2	**<10** ^**−6**^
Age, years	43.0 ± 12.3	45.6 ± 10.7	47.9 ± 12.3	0.2340
Age of onset, years	20.9 ± 14.2	23.4 ± 15.5	26.1 ± 18.3	0.4040
Body Mass Index (kg/m^2^)	25.7 ± 3.6	27.3 ± 3.7	26.8 ± 4.2	0.2308
Percent of clinical cluster group				
Mild, no ICS (%)	na	na	na	
Moderate, low-medium ICS (%)	62%	58%	29%	**0.0135**
Severe, high ICS (%)	38%	42%	71%	
Male (%)	49%	44%	48%	0.9240
Atopic, Phadiatop positive (%)	92%	72%	71%	0.0530
Sputum Pauci (Eos < %3, PMN < 60%) (%)^b^	10%	38%	22%	
Sputum Neutr. (Eos < 3, PMN > =60%) (%)^b^	14%	46%	17%	**0.0003**
Sputum Mix (Eos > =3%, PMN > =60%) (%)^b^	14%	8%	30%	
Sputum Eos. (Eos > =3%, PMN < 60%) (%)^b^	62%	8%	30%	
FENO ≥ 35 ppb (%)	59%	3%	43%	**1.8 x10** ^**−6**^
Blood eosinophils ≥ 300/ul (%)	54%	6%	32%	**4.1 x10** ^**−5**^
FENO ≥ 35 ppb OR Blood eos. ≥ 300/ul (%)	81%	8%	55%	**<10** ^**−6**^
Biopsy CCL26-high (%)^c^	33%	11%	29%	0.2179

^a^Summary statistics (mean, or geometric mean where indicated, and standard deviation; or percent of clinical cluster group) and *p*-values from F-test (for mean/geometric mean statistics) or chisquare statistics for associations among the ADEPT clinical cluster groups, restricted to moderate-severe asthma subjects and excluding Group 1, are presented. The 9 clinical and clinical biomarker variables used in the clustering are presented first, followed by additional clinical, biomarker and demographic variable groupings

^b^For sputum differentials, *n* = 29, 24, and 23 for Groups 2, 3, and 4, respectively

^c^For endobronchial biopsy gene expression variables (from Affymetrix HG-U133 + PM array signal intensities), *n* = 21, 20, and 14 for Groups 2, 3, and 4, respectively

Significant *p* values are captured in bold

### The ADEPT phenotypes resist perturbation

Sensitivity analyses were performed to confirm robustness of the clusters to perturbations of removing groups of subjects. In the first sensitivity analysis, only 1 of 152 subjects changed cluster assignment after excluding the ‘medoid’ subject defining each cluster group. Next, only 4 of 104 subjects from original clusters 2, 3, and 4 changed cluster assignment after the mild asthma cohort was excluded, with 3 clusters selected for analysis. Similarly, when the moderate asthma cohort was excluded, only 4 of 101 subjects changed cluster assignment. Only when the severe cohort was excluded was there a marked deterioration in cluster formation, with 37 of 105 changing cluster assignment, specifically with Group 4 not well-forming in this scenario. This was not unexpected given that the original Group 4 consisted mostly of severe asthma subjects.

### The ADEPT phenotypes are stable longitudinally

**Fig. 5:**
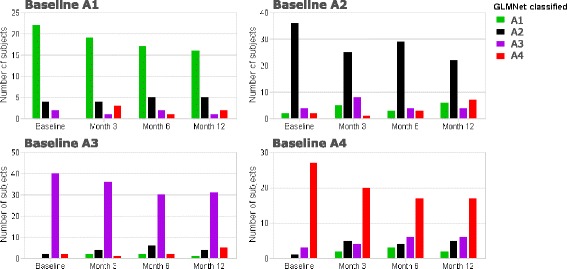
Longitudinal evaluation of ADEPT-asthma clinical phenotype classification. GLMnet-classification model of ADEPT-asthma baseline clinical phenotypes (7 clustering variables, excluding PC20 and blood eosinophils; Model B) was applied to classify the ADEPT-asthma participants based on data from the baseline and 3, 6, and 12 month follow-up visits. Each panel presents ADEPT asthma participants assigned to the indicated clinical phenotypes from the baseline clustering analysis, reporting the phenotype to which they are classified at the indicated follow-up visits

Applying “Model B” (excluding blood eosinophils and PC_20_) to classify ADEPT participants for the baseline, 3, 6 and 12-month visits, stratified by the original baseline clustering assignment, 24 of 147 participants (16%) had a discordant baseline classification relative to their originally assigned baseline phenotype (i.e., 16% error rate in classification performance). By comparison using Model A (including blood eosinophils but not PC_20_) to classify baseline samples, discordance was reduced to a 4% error in classification to the originally assigned phenotype (Fig. 2). Fig. 5 displays the frequency of subjects that maintain or change phenotype classification across the longitudinal visits. In general, the majority of participants had stable phenotype assignments throughout the 12-month duration of the study. The Pearson’s Contingency Coefficients representing the within-subject consistency of classified phenotype across visits were 0.81, 0.74, 0.76, and 0.78 for actual baseline phenotypes A1, A2, A3, and A4, respectively. The overall discordance for classified vs. originally assigned phenotypes was 31%, 31%, and 40% for the 3, 6, and 12 month visits (compared to the baseline discordance error rate of 16%) (Fig. 5). Original baseline Phenotype A4 showed the most changes at follow-up visits, with 39%, 40%, and 40% of participants with discordant classifications at the 3, 6, and 12 month visits, respectively. Additional file 1: Figure S1 shows how subjects changed cluster classifications over time for subjects having discordant classifications for at least one-time point compared to the original, baseline clustering assignment. Case-reports for associated changes in clinical and biomarker variables are described in Additional file 1: Figure S2 for subjects having discordant classifications compared to baseline (one random subject per baseline cluster selected for presentation) and in Additional file 1: Figure S3 for subjects having concordant classifications across visits compared to baseline assignment (one random subject per baseline cluster selection for presentation).

### The ADEPT matched U-BIOPRED sub-population also optimally partitions into 4 phenotypes

**Fig. 6:**
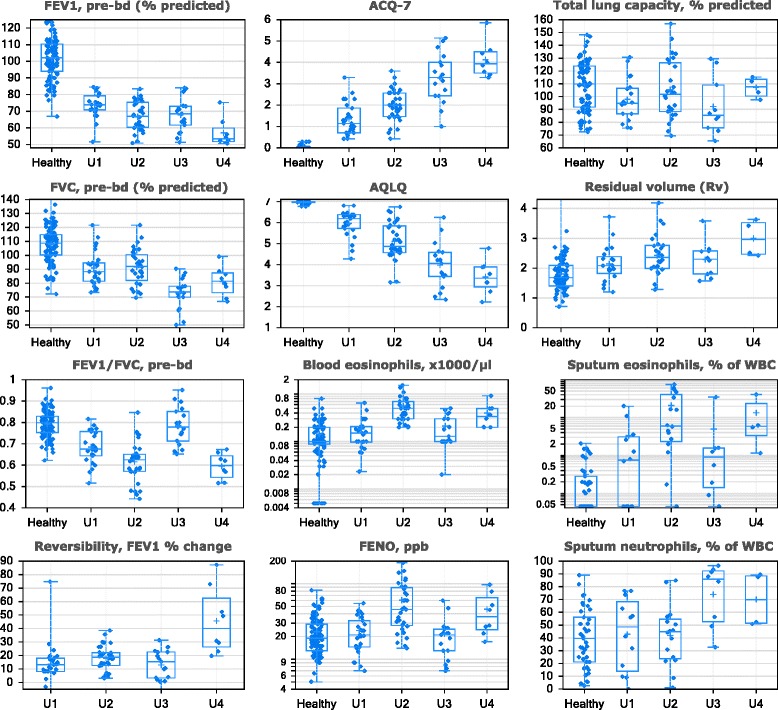
Clustering variables, sputum granulocytes, and plethysmography in U-BIOPRED clinical phenotypes. The values (y-axis) for the indicated variables (indicated at top of the plot) are shown for U-BIOPRED asthma participants stratified by fuzzy-PAM clinical phenotypes (x-axis). Data presented as symbols representing individual participants and summarized by box (inter-quartile range and median) & whiskers (range), with ‘+’ indicating mean. Pre-bd, pre-bronchodilator; WBC, white blood cells

For the U-BIOPRED cohort [11], 82 out of 397 participants were selected for clustering based on similar clinical inclusion criteria as ADEPT participants excluding U-BIOPRED participants on OCS as well as smokers. When compared to ADEPT moderate-severe asthmatics, the restricted U-BIOPRED set had slightly lower FEV_1_ (mean ± standard deviations of 71.9 ± 10.7 vs. 68.1 ± 9.8, *p* = 0.011), FVC (91.6 ± 11.6 vs. 86.5 ± 14.1, *p* = 0.007), and AQLQ (5.4 ± 1.2 vs. 5.0 ± 1.2, *p* = 0.018) and higher ACQ-7 (1.6 ± 0.9 vs. 2.3 ± 1.2, *p* = 0.000045). This also resulted in an optimum of 4 phenotypes, based on Fc(U)/Dc(U) partition metrics of 0.776/0.060, 0.810/0.054, 0.837/0.049 (optimal), and 0.800/0.080, for options with 2, 3, 4, and 5 clusters, respectively. The U-BIOPRED phenotypes, US (U-BIOPRED subset)-1 to US4, shared similar characteristics as A1, A2, A3 and A4, respectively. More than 75% of participants in each cluster had >81% probability for their assigned phenotype, and only 2 participants (in Phenotype US1 and US2) had <50% probability of belonging to their phenotype (Fig. 2). All clustering variables were significantly associated across the 4 phenotypes. Figure 6 and Table 3 show clustering, clinical, biomarker, and demographic variables for the US1 to US4 clusters.

**Table 3:** Clinical, biomarker, and demographic associations among the U-BIOPRED adult asthma clinical clusters

Variable^a^	Healthy (*n* = 101)	Cluster US1 (*n* = 25)	Cluster US2 (*n* = 32)	Cluster US3 (*n* = 17)	Cluster US4 (*n* = 8)	*P*-value
Mean [Geo.Mean] ± SD						
FEV1, % predicted (pre-bd)	101.8 ± 12.9	73.7 ± 7.3	66.6 ± 9.0	67.6 ± 9.9	57.0 ± 8.5	**8.8 x10** ^**−5**^
FVC, % predicted (pre-bd)	107.8 ± 13.4	90.9 ± 12.4	91.5 ± 12.6	72.9 ± 11.5	81.2 ± 10.5	**6.1 x10** ^**−6**^
FEV1/FVC (pre-bd)	0.79 ± 0.06	0.68 ± 0.08	0.62 ± 0.09	0.79 ± 0.09	0.60 ± 0.06	**<10** ^**−6**^
bd reversibility, % change FEV1	na	13.7 ± 16.7	17.9 ± 9.3	12.9 ± 12.5	45.6 ± 24.5	**3.1 x10** ^**−6**^
FENO, ppb [Geo.Mean]	19.9 + 14.2/-8.3	20.8 + 16.0/-9.1	47.4 + 49.8/-24.3	18.1 + 15.0/-8.2	38.8 + 33.4/-17.9	**1.6 x10** ^**−6**^
ACQ7	0.0 ± 0.1	1.4 ± 0.7	2.0 ± 0.7	3.3 ± 1.2	4.1 ± 0.8	**<10** ^**−6**^
AQLQ	7.0 ± 0.1	6.0 ± 0.6	5.1 ± 0.9	4.0 ± 1.1	3.4 ± 0.8	**<10** ^**−6**^
Blood eosinophils, x1000/ul [Geo.Mean]	0.098 + 0.180/-0.063	0.143 + 0.155/-0.075	0.484 + 0.356/-0.205	0.161 + 0.204/-0.090	0.345 + 0.254/-0.146	**<10** ^**−6**^
Blood neutrophils, ×1000/ul [Geo.Mean]	3.23 + 1.67/-1.10	3.77 + 1.58/-1.11	4.09 + 1.33/-1.00	4.57 + 2.13/-1.45	1.84 ± 0.49	0.2128
Blood WBC, ×1000/ul [Geo.Mean]	5.64 + 2.04/-1.50	6.41 + 1.62/-1.29	7.39 + 1.79/-1.44	7.83 + 2.24/-1.74	2.71 ± 0.36	**0.0236**
Sputum neutrophils, % of WBC^b^	30.8 + 40.3/-17.4	42.8 ± 28.3	42.0 ± 23.5	73.9 ± 24.2	70.0 ± 21.3	**0.0129**
Sputum eosinophils, % of WBC [Geo.Mean]^b^	0.2 + 0.3/-0.1	0.5 + 4.7/-0.5	6.1 + 44.5/-5.4	0.7 + 4.8/-0.6	6.3 + 21.0/-4.9	**0.0123**
IgE Total (IU/ml) [Geo.Mean]	25 + 87/-19	121.1 + 377.2/-91.7	245.0 + 1,005.0/-197.0	84.5 + 267.5/-64.2	179.6 + 295.1/-111.7	0.0940
Age	39 ± 13	46 ± 15	49 ± 16	50 ± 12	44 ± 15	0.7000
Age of onset, years	na	21 ± 18	26 ± 19	29 ± 19	14 ± 12	0.2028
Body Mass Index (kg/m2)	25.3 ± 3.6	27.6 ± 5.4	27.6 ± 5.8	30.9 ± 7.8	27.1 ± 4.8	0.2408
Percent of clinical cluster group						
Severe / Non-severe asthma (N / N)	na	11 / 14	25 / 7	16 / 1	8 / 0	**0.0004**
Male (%)	61%	36%	34%	29%	25%	0.9276
Atopic, Skin prick test or serum IgE-RAST (%)	45%	100%	86%	88%	100%	0.5816
Sputum Pauci (Eos < %3, PMN < 60%) (%)^b^	76%	42%	25%	25%	0%	**0.0496**
Sputum Neutr. (Eos < 3, PMN > =60%) (%)^b^	24%	33%	6%	63%	25%	
Sputum Mix (Eos > =3%, PMN > =60%) (%)^b^	0%	0%	6%	0%	25%	
Sputum Eos. (Eos > =3%, PMN < 60%) (%)^b^	0%	25%	63%	13%	50%	
FENO ≥ 35 ppb (%)	18%	24%	59%	12%	50%	**0.0032**
Blood eosinophils ≥ 300/ul (%)	7%	20%	75%	35%	63%	**0.0003**
FENO ≥ 35 ppb OR Blood eos. ≥ 300/ul (%)	21%	32%	88%	41%	88%	**3.3 x10** ^**−5**^

^a^Summary statistics (mean, or geometric mean where indicated, and standard deviation; or percent of clinical cluster group) and *p*-values from F-test (for mean/geometric mean statistics) or chi-square statistics for associations among the U-BIOPRED adult asthma clinical cluster groups are presented. The 8 clinical and clinical biomarker variables used in the clustering are presented first, followed by additional clinical, biomarker and demographic variable groupings

^b^For sputum differentials, *n* = 12, 16, 8, and 4 for the healthy control cohort and Groups A, B, and C, and D, respectively

Significant *p* values are captured in bold

**Phenotype US1 (‘mild, good lung function, early onset, low inflammation’)** was the least symptomatic, with modest reversible obstruction and good asthma control. Eosinophilic inflammation was minimal (see Table 3: FENO, blood eosinophils). US1 was most similar to A1, albeit with a greater BDR and lower FENO levels, the latter perhaps due to ICS treatment in US1 but not A1 participants. US1 was generally more severe than A1.

**Phenotype US2 (‘moderate, hyper-responsive, eosinophilic’)** in general is characterized by reversible obstruction, uncontrolled asthma, normal FVC, and predominantly eosinophilic inflammation, with 88% of participants being FENO-high (≥35 ppb) or blood eosinophil-high (≥300/μL). For those with induced sputum, most (69%) had high sputum eosinophils (≥3% of leukocytes) and few (12%) had high sputum neutrophils (≥60% of leukocytes). Phenotype US2 was largely homologous to phenotype A2.

**Phenotype US3 (‘mixed severity, mild reversible obstruction, non-eosinophilic, neutrophilic’)** in general had moderately reduced FEV1, reduced mean FVC (72.9 ± 11.5%% predicted), poor asthma control, and low-to-modest BDR. Phenotype US3 was neutrophilic and non-eosinophilic, with only 41% of participants being FENO-high or blood eosinophil-high, compared to 21% observed in the healthy control group. The majority of participants (63%) were sputum neutrophil-high, and few (12.5%) were sputum eosinophil-high. Phenotype US3 was, therefore, largely homologous to Phenotype A3.

**Phenotype US4** (**‘severe uncontrolled, severe reversible obstruction, mixed granulocytic’)** in general was characterized by marked airflow obstruction, high BDR with reduced FVC, and very poor asthma control. These participants were eosinophilic, with 88% of the phenotype being FENO-high or blood eosinophil-high and 75% having elevated sputum eosinophils, but 50% also having high sputum neutrophils. Phenotype US4 was homologous to Phenotype A4.

Plethysmography data were available in the U-BIOPRED study (Additional file 1: Table S5). Participants in Phenotype US4 had relatively normal total lung capacity but elevated residual volumes (67% higher than HV), and 24 – 40% higher than Phenotypes US1, US2, and US3. These results indirectly suggest that the reduced FVC (mean 81% predicted) was a consequence of air trapping for Phenotype US4.

The U-BIOPRED study included several patient-reported outcome (PRO) questionnaires not included in the ADEPT study: Epworth sleepiness scale, Hospital Anxiety and Depression Scale (HADS), Medication Adherence Report Scale (MARS), Sino-Nasal Outcome Test (SNOT), (Additional file 1: Table S5). Phenotypes US1, US3, and US4 subjects had higher scores for Epworth sleepiness scale compared to healthy participants (*p* < 0.05). Except for phenotype US1, HADS scores were elevated in each cluster compared to healthy participants (*p* < 0.05). All 4 clusters had significantly higher SNOT scores (*p* < 7x10^−5^), particularly for clusters US3 and US4. Importantly, all 4 U-BIOPRED phenotypes had similar scores on the MARS questionnaire, suggesting that clinical and biomarker differences between the clusters is not largely driven by differential adherence to medication usage.

### Classification of U-BIOPRED participants to the ADEPT baseline phenotypes

**Fig. 7:**
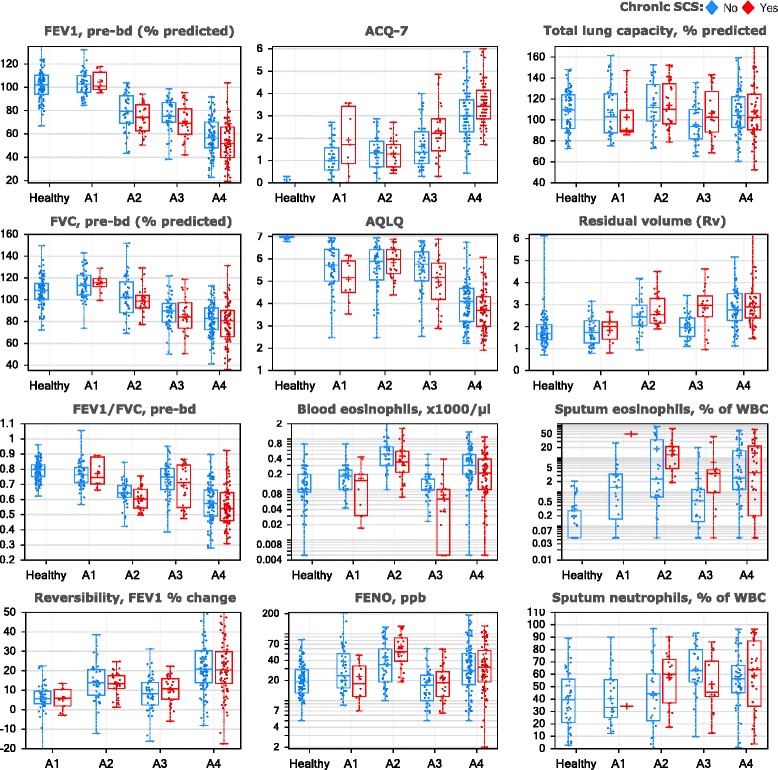
Clustering variables in U-BIOPRED participants classified to ADEPT clinical phenotypes. The values (y-axis) for the indicated variables (indicated at top of plot) are shown U-BIOPRED healthy controls and asthma participants classified to ADEPT clinical phenotypes (Model A) (x-axis), stratified chronic systemic corticosteroid (SCS) use (blue, no; red, yes). Data presented as symbols representing individual participants and summarized by box (inter-quartile range and median) & whiskers (range), with ‘+’ indicating mean. Pre-bd, pre-bronchodilator; WBC, white blood cells

As an alternate strategy to assess how well the ADEPT clinical cluster structure fits to an independent study population, GLMnet-classification model of ADEPT-asthma baseline clinical clusters (8 clustering variables, excluding PC20; Model A) was applied to classify the subset of 397 participants from the U-BIOPRED asthma subjects (not restricted to ADEPT inclusion criteria and for whom all clustering clinical data were available). The classification probabilities for the classified ADEPT clusters are reported in Fig. 2 for both ADEPT (panel c, using baseline data to demonstrate model performance) and U-BIOPRED (panel D), with stratification by systemic steroid use for U-BIOPRED. For ADEPT-asthma, the classification concordance to actual clusters was excellent at 96% overall for all clusters. When applying the classification model to U-BIOPRED, the probability distributions were similar to that in ADEPT. Remarkably, the maintenance OCS group, not represented in ADEPT, also fitted well into ADEPT phenotype classification structure, with most participants having >80% probability for their classified cluster. Importantly, the relative distributions of the 8 clustering variables were similar across the 4 classified ADEPT clinical phenotypes for U-BIOPRED participants (Fig. 7) compared to ADEPT asthmatic participants (Fig. 3) even for participants taking or not taking chronic OCS.

### Airway T2 characteristics of asthma phenotypes

In addition to the clinical and clinical biomarker ‘clustering’ variables assessed, plus sputum differential cell counts, biopsy gene expression was available in 81 of 156 subjects included in the ADEPT clustering. An active airway T2 phenotype was defined as the observed interleukin (IL)-13 activity in endobronchial samples, indicated by the gene expression of IL-13-inducible genes. For the ADEPT study, this was specifically evaluated by gene expression of CCL26 in endobronchial biopsies. Despite having the 2^nd^ lowest levels of FENO, blood eosinophils, and sputum eosinophils, Phenotype A1 had the highest proportion of participants (56%) who had high CCL26 gene expression in their endobronchial biopsies (CCL26-high asthmatics defined as beyond the 95^th^ percentile of healthy control distribution; Additional file 1: Section S5), followed by Phenotype A2, which had slightly fewer (41%) CCL26-high participants (Table 1). Consistent with being the least eosinophilic, Phenotype A3 had the least (16%) CCL26-high participants (Table 1). Phenotype A4 was intermediate, with 32% CCL26-high participants. A similar pattern among the phenotypes was observed for POSTN gene expression as an additional indicator of T2 phenotype (data not shown).

Too few of the 82 U-BIOPRED asthma participants included in the clustering had endobronchial biopsy or brushings samples available for analysis (*n* = 16 and 17, respectively). For the 77 of 397 U-BIOPRED participants classified to ADEPT clusters who had available data on microarray gene expression in epithelial brushings, the T2 distributions (defined by IL-13 in vitro stimulation signature enrichment) were similar to those observed for ADEPT participants despite the differences in tissue and indicators for defining T2 status (Data not shown and Additional file 1: Figure S4), with Phenotypes US2 and US4 having the highest proportions of T2-high participants (38%) and Phenotype US3 having the fewest (6%). Only the U-BIOPRED Phenotype US1 had lower proportions of T2-high participants (21%) compared to Phenotype A1 (56%), perhaps a consequence of ICS use in the U-BIOPRED but not ADEPT asthmatics in Phenotype A1.

## Discussion

To our knowledge, this is the first study to identify reproducible phenotypes of asthma using data from two entirely independent cohorts of asthmatic patients. The four observed phenotypes have clinical relevance, representing patient subtypes that are commonly encountered in clinical practice, namely mild, well-controlled asthmatics, moderately-controlled asthmatics with severe AHR, asthmatic with less reversible airways disease, and severe asthmatics. The large size of the study, enrolling participants from across the EU, US and Canada, and the use of clinical variables that can be obtained easily in any pulmonary center provides a valuable novel methodological tool for asthma research.

Initial clustering of the ADEPT study dataset resulted in four phenotypes that were robust to perturbation and generally stable over one year. These phenotypes were further validated in a subset of the U-BIOPRED cohort dataset, where four remarkably analogous phenotypes were observed. Importantly, differential biology across phenotypes was observed which will help develop tailored therapeutic options. Of relevance to current novel therapies that target T2 immune mechanisms, the phenotypes could be differentiated in respect of expression of gene sets that are characteristic of the pathobiology driven by the T2 cytokine, IL-13. Phenotype A1, was remarkably similar to Phenotype US1 from the U-BIOPRED validation set. The low degree of airway inflammation in A1 was commensurate with absence of a decision to start controller medications, while in the US1 phenotype, where all the asthmatics were on ICS, this suggested adequate suppression of airway inflammation and, consequently, good disease control. Of note, the A1 phenotype had the highest proportion of T2 high asthmatics even though their levels of exhaled NO, proposed as good biomarkers of T2 inflammation [20], were relatively low. This suggests that T2 inflammation, on its own, may not be a determinant of clinical severity. Given the good clinical characteristics of this phenotype, there seems to be minimal need for new therapies, although this phenotype could be targeted for disease interception.

The asthmatics in Phenotype A2, based on their ACQ-7 scores (mean 1.1), were between the cut-off for good (<0.75) and poor control (>1.5), suggesting less than optimal control. With respect to underlying pathobiology, their gene expression profile was highly skewed towards T2 inflammation. Consistent with previous reports in mild, steroid-naive asthmatics [21], the high expression of IL-13 induced genes was associated with the highest eosinophilic profile amongst all four phenotypes. The preserved lung function and asthma control, but severe AHR may represent “brittle” asthma, a recognized patient phenotype. Given that these asthmatics were on regular treatment with corticosteroids, the high T2 profile suggests a significant degree of steroid insensitivity; therefore, treatment with biologics or other drugs targeting T2 inflammation might be indicated in this phenotype to achieve improved control.

Phenotype A3 had reduced lung capacity and lower BDR (compared to A2 and A4), which could suggest more extensive tissue remodeling. The asthmatics in this phenotype tended to be more neutrophilic and could, therefore, be viewed as having phenotypic similarities to COPD, with disease potentially driven more by infection than atopy. Consistent with the neutrophilic, rather than eosinophilic, nature of the disease, T2 gene expression was the lowest of all phenotypes. While not studied in ADEPT, the role of the microbiome in the airway or GI tract might be important, supporting evaluation of alternative anti-inflammatories e.g. macrolide antibiotics in this phenotype [22]. This phenotype has few therapeutic options at present so further study of the underlying mechanisms and how these impacts on clinical expression are needed.

Of all the observed phenotypes, Phenotype A4 was the most severe, with ACQ (mean 2.6) well above the cut-off for poor control and with 63% of participants classified as severe on enrollment. These asthmatics had the worse mean FEV_1_ (66%) and were severely hyper-responsive. Their sputum had a mixed granulocytic profile, with both neutrophil and eosinophil counts being high. Of importance, they had an airway T2-high profile, despite treatment with ICS, which could, like A2, represent relative steroid insensitivity. Patients with this profile are the commonest participants included in recent asthma studies for novel T2-inflammation focused therapeutics, e.g. anti-IL-13 and anti- IL-5 monoclonal antibodies [23, 24]. These asthmatics had the second highest concentrations of FENO. Thus, those asthmatics in this phenotype with high levels of predictive biomarkers such as eosinophilia and FENO may well respond to therapies targeting IL-13 biology and/or eosinophilic inflammation [23, 25]. Alternatively, high FENO concentrations could represent poor adherence to treatment with ICS [26].

There are many possible influences on stability of phenotypes such as allergen exposure, air pollution, as well as emergent changes in asthma medications, and viral infections. Thus, it is interesting that ADEPT participants remained relatively stable for their phenotype assignments throughout the 12-month duration of the study, suggesting that the phenotypes represent different disease driving biological mechanism but their specific combinations within a phenotype remain stable over time. Phenotype A4 was the least stable of the groups, and we would speculate that periods of reduced environmental triggers could improve control in this most severe group, resulting in a shift to Phenotype A2. Alternatively, improved responsiveness to inhaled steroids with resulting reduction in T2 inflammation (whether environmentally based or from improved adherence during the study) could result in a shift to Phenotype A3. However, the appropriate data to support such possibilities are not available and thus such explanations remain speculative.

This study makes valuable observations when comparing two entirely independent cohorts where participants are not matched a priori. Participants in the ADEPT study were selected on the basis of steroid therapy and significant obstruction (FEV_1_ ≤ 80% of predicted), in order to match standard interventional clinical trial enrollment criteria. At the same time, more restrictive limits were placed on other clinical parameters such as BMI (≤32 kg/m^2^) and smoking asthmatics were excluded. U-BIOPRED had a much broader representation of the overall asthmatic population, including severe asthmatics on maintenance OCS treatment and much lower FEV_1_. Because of these differences, the U-BIOPRED primary validation set only included those participants most similar to those in ADEPT. Although PC_20_ data were not available for most of the U-BIOPRED participants, the four ADEPT phenotypes largely mapped to the resultant 4 U-BIOPRED phenotypes for clinical variables. However, when applying the ADEPT classification model to a larger subset of the adult participants in U-BIOPRED cohort, the asthmatics on maintenance OCS and smoking-associated severe asthma also fitted well into the ADEPT phenotype classification structure, with similar relative distributions of the 8 clustering variables across the 4 classified ADEPT clinical phenotypes.

When considering the underlying biology, there was substantial homology between Phenotypes A1, A2, A3, and A4 and Phenotypes US1, US2, US3 and US4, respectively, with similar homology for most inflammatory variables. In comparison to Phenotype A2, the equivalent US2 phenotype from U-BIOPRED, had very similar bronchodilator responsiveness, albeit slightly worse pre-bronchodilator FEV_1_ (mean 66%). The T2 profile in Phenotype US2 was similarly skewed towards T2. Both Phenotypes A3 and US3 shared features with COPD (lower BDR compared to A2 and A4, low FENO, and neutrophilia). However, despite relatively low eosinophilic inflammation in both A1 and US1, FENO and airway T2-high status tended to be higher in A1 but low in US1, likely a consequence of ICS requirement in U-BIOPRED but not in ADEPT. This observation suggests that FENO levels may be more directly associated with lower airway T2 activity than eosinophilic inflammation per se. We have observed that the T2-high phenotype is a subset of a broader eosinophilic phenotype group in ADEPT and U-BIOPRED studies [27], consistent with results from Choy et al. [28] in mild asthma (evaluating T2 phenotype status by POSTN, SERPINB2, and CLCA1 expression in endobronchial brushings).

T2 inflammation is a major characteristic defining the clusters, but mainly distinguishes Phenotype A3 (low T2 inflammation) from the other 3 phenotype groups. Phenotypes A1, A2, and A4 are then clinically distinguished by degree of asthma control and airflow limitations. Ideally T2 and eosinophilic inflammation would be established from airway mucosal and sputum sampling, but these are difficult to evaluate in a standard clinical setting. Blood eosinophils, FENO, and potentially serum proteins could serve as surrogates to establish these phenotypes, but alone are insufficient to define such. Combinations of the surrogates, as utilized in the classification of the phenotype groups, are needed for more accurate estimation of T2 and eosinophilic phenotypes.

The variables selected for clustering in this study were different from those in previous reports (Additional file 1: Table S1), applying variables readily measured in clinical practice and trials that represent the current clinical presentation of asthma, as opposed to demographic and historical aspects of the disease. Likewise, although gender and smoking history may be associated with disease, they do not directly represent the pathology of asthma. Post-hoc perturbation analyses (not reported) assessing the impact of adding in additional demographic, historical, and treatment attributes (e.g., gender, age, atopy, asthma age-of-onset, BMI, ICS dose; 20 variables total), resulted in weaker clustering (based on distance metrics and cluster membership probabilities), with qualitatively similar clinical associations as reported for the ADEPT asthma phenotypes (65% concordance of the most closely analogous clusters with the reported ADEPT asthma phenotypes). FENO was an influential variable in the clustering. Inclusion of FENO could potentially indicate steroid insensitivity and/or a basal non-inflammatory phenotype. Given that FENO and blood eosinophil levels were not higher in cluster A1 (mostly no ICS) compared to clusters 2 and 4 (low-medium and high ICS), FENO and blood eosinophil levels are not simply an indicator of ICS use. Compliance was not formally monitored in these studies. However, suppression of B cell and T cell lineage gene expression in biopsies below levels in healthy controls and mild asthmatics (not taking ICS), and suppression of mast cell lineage gene expression below that in mild asthma (data not shown), suggests that most subjects in the moderate and severe asthma groups were compliant in taking ICS, at least around the time of biopsy. The fitting of the U-BIOPRED severe asthmatics on oral CS to the clusters furthers confidence that the cluster formations were not overly influenced by lack of compliance to steroids. Nevertheless, even in the best of circumstances, compliance can only be evaluated over the short-term and imperfectly in absence of inpatient-monitoring.

The strength of this study is represented by the independent, external validation and the longitudinal stability assessment. Nevertheless, there are also limitations. The actual prevalence of the clinical clusters cannot be directly estimated because participants were not randomly recruited from the general asthma population. ADEPT had strict inclusion/exclusion criteria to restrict enrollment to patients likely to be enrolled in interventional clinical studies. Thus morbidly obese participants, smoking participants and chronic OCS-treated participants are not represented in ADEPT. In contrast, U-BIOPRED did include smokers and chronic OCS treated patients, with almost 50% of asthmatics on chronic OCS therapy. These features in fact predominated in the distinction of the 4 clinical clusters that have been reported with the U-BIOPRED cohort [29] (Additional file 1: Section S12). Indeed, 4 clusters were identified, of which the first consisted of well-controlled mild-to-moderate asthmatics, while the 3 other clusters consisting of predominantly severe asthma patients were characterised by 2 clusters of chronic airflow obstruction, one with late-onset asthma in predominant smokers/ex-smokers with high BMI and the other in non-smokers with high OCS use; the fourth cluster consisted of predominantly obese female patients with uncontrolled asthma and increased exacerbations, but with normal lung function [29].

## Conclusion

In summary, we have provided evidence for four phenotypes that are stable over time and are differentiated by both clinical severity, response to their prescribed treatment and the underlying T2 gene expression profile. Critically, these phenotypic groups were validated in an independent asthma cohort. Further extensive gene expression data and other ‘omics’ analyses remain available for more in-depth evaluation of molecular profiles associated with these newly defined clinical phenotypic groups. Focusing on the biology of each phenotype and understanding the unmet need will aid in developing tailored therapies.

## Additional file

Additional file 1: Table S1. List of clustering variables for several studies. **Table S2.** Clinical and demographic characteristics of ADEPT and U-BIOPRED severity cohorts. **Table S3.** Linear predictor function coefficients for classification to ADEPT-asthma baseline clinical clusters. **Table S4.** IL-13 in vitro stimulation (IVS) signature. **Table S5.** Plethysmography and PRO variable associations among the U-BIOPRED adult asthma clinical clusters. **Figure S1.** (A). Longitudinal ADEPT cluster classifications discordant with baseline Cluster A1. **Figure S1.** (B). Longitudinal ADEPT cluster classifications discordant with baseline Cluster A2. **Figure S1.** (C). Longitudinal ADEPT cluster classifications discordant with baseline Cluster A3. **Figure S1.** (D). Longitudinal ADEPT cluster classifications discordant with baseline Cluster A4. **Figure S2.** Characterization of selected subjects changing ADEPT cluster classifications. **Figure S3.** Characterization of selected subjects with stable ADEPT cluster classifications. **Figure S4.** IL-13 activity in U-BIOPRED participants classified to ADEPT clinical clusters. (XLSX 461 kb)

## Abbreviations

ACQ7: Asthma control questionnaire 7 (include FEV1)
ADEPT: Airways Disease Endotyping for Personalized Therapeutics
AHR: Airways hyperresponsiveness
AQLQ: Asthma quality of life questionnaire
BDR: Bronchodilator reversibility
bEOS: Blood eosinophils
CCL: C-C motif chemokine ligand
EBBX: Endobronchial biopsy
EOS: Eosinophils
EPBR: Epithelial brushings
FEF25-75: Forced expired flow averaged between 25–75% of the vital capacity
FENO: Fractional concentration of exhaled nitric oxide
FEV1: Forced expired volume in 1 second
FVC: Forced vital capacity
GSVA: Gene set variation analysis
HV: Healthy Volunteers
ICS: Inhaled corticosteroids
IFN: Interferon
IgE: Immunoglobulin E
IL: interleukin
IL-4R: Interleukin 4 receptor
IS: Induced sputum
IVS: In-vitro signature
LOQ: Limit of quantification
MAB: Monoclonal antibody
Mod-Sev: Moderate-severe asthma (combined)
mRNA: Messenger ribonucleic acid
NHANES: National Health and Nutrition Examination Survey
PC20: The provocative concentration of methacholine resulting in a 20% or greater fall in the forced expired volume in 1 s
PN: Predicted normal
RNA: Ribonucleic acid
SARP: Severe Asthma Research Program
SCS: Systemic corticosteroids
spEOS: Sputum eosinophils
SST: Serum separation tubes
Th2: T helper cell type 2
TLC: Total lung capacity
U-BIOPRED: Unbiased Biomarkers for the Prediction of Respiratory Disease Outcome Consortium
